# Synthesis, characterization and biocompatibility of cadmium sulfide nanoparticles capped with dextrin for in vivo and in vitro imaging application

**DOI:** 10.1186/s12951-015-0145-x

**Published:** 2015-11-17

**Authors:** Jorge Reyes-Esparza, Alberto Martínez-Mena, Ivonne Gutiérrez-Sancha, Patricia Rodríguez-Fragoso, Gerardo Gonzalez de la Cruz, R. Mondragón, Lourdes Rodríguez-Fragoso

**Affiliations:** Facultad de Farmacia, Universidad Autónoma del Estado de Morelos, Cuernavaca, 62210 Mexico; Departamento de Física, CINVESTAV-I.P.N., Apartado Postal 14-740, 07000 Mexico, D.F. Mexico; Departamento de Bioquímica, CINVESTAV-I.P.N., Apartado Postal 14-740, 07000 Mexico, D.F. Mexico

**Keywords:** Quantum dot, Cadmium sulfide nanoparticles, Cytotoxicity, Apoptosis, Biocompatibility biodistribution

## Abstract

**Background:**

The safe use in biomedicine of semiconductor nanoparticles, also known as quantum dots (QDs), requires a detailed understanding of the biocompatibility and toxicity of QDs in human beings. The biological characteristics and physicochemical properties of QDs entail new challenges regarding the management of potential adverse health effects following exposure. At certain concentrations, the synthesis of semiconductor nanoparticles of CdS using dextrin as capping agent, at certain concentration, to reduce their toxicity and improves their biocompatibility.

**Results:**

This study successfully synthesized and characterized biocompatible dextrin-coated cadmium sulfide nanoparticles (CdS-Dx/QDs). The results show that CdS-Dx/QDs are cytotoxic at high concentrations (>2 μg/mL) in HepG2 and HEK293 cells. At low concentrations (<1 μg/mL), CdS-Dx/QDs were not toxic to HepG2 or HeLa cells. CdS-Dx nanoparticles only induced cell death by apoptosis in HEK293 cells at 1 μg/mL concentrations. The in vitro results showed that the cells efficiently took up the CdS-Dx/QDs and this resulted in strong fluorescence. The subcellular localization of CdS-Dx/QDs were usually small and apparently unique in the cytoplasm in HeLa cells but, in the case of HEK293 cells it were more abundant and found in cytoplasm and the nucleus. Animals treated with 100 μg/kg of CdS-Dx/QDs and sacrificed at 3, 7 and 18 h showed a differential distribution in their organs. Intense fluorescence was detected in lung and kidney, with moderate fluorescence detected in liver, spleen and brain. The biocompatibility and toxicity of CdS-Dx/QDs in animals treated daily with 100 μg/kg for 1 week showed the highest level of fluorescence in kidney, liver and brain. Less fluorescence was detected in lung and spleen. There was also evident presence of fluorescence in testis. The histopathological and biochemical analyses showed that CdS-Dx/QDs were non-toxic for rodents.

**Conclusions:**

The in vitro and in vivo studies confirmed the effective cellular uptake and even distribution pattern of CdS-Dx/QDs in tissues. CdS-Dx/QDs were biocompatible with tissues from rodents. The CdS-Dx/QDs used in this study can be potentially used in bio-imaging applications.

## Background

Biomedical applications exploit the fluorescent properties of quantum dots (QDs), particularly their advantage over traditional organic dyes, for both diagnostic and clinical applications [1]. QDs are also being researched for use in whole-body in vivo imaging of normal and tumor tissues. QDs may also have a use in therapeutic applications such as targeted drug delivery, photodynamic therapy, and drug discovery [2–4].

The incorporation of QDs into biological systems often requires strategies for the manipulation of the ligands bound to the surface of the QDs surface in order to make them water-soluble and biocompatible–that is, compatible with living tissues or a living system by being neither toxic nor injurious or physiologically reactive [5]. QDs must be rendered water-soluble through the modification of their surface in preparation for biological applications. However, high-quality QDs are mainly made with heavy metals like cadmium, the long-term toxicity of which is currently largely unknown.

Cadmium, which is the main component in the majority of QDs, is known to be acutely and chronically toxic to cells and organisms. In cells, it is taken into calcium membrane channels, where it accumulates [6–8]. Its toxicity to living organisms is mainly associated with liver and kidney injury, osteoporosis and neurological dysfunctions [9]. Protecting the core can, to some degree, control toxicity related to cadmium leakage. However, the change in the physicochemical and structural properties of engineered QDs could be responsible for a number of material interactions that could also have toxicological effects [10, 11].

The stability of cadmium-containing QDs in water can be obtained through either a complete ligand exchange procedure, or through steric stabilization, where the native hydrophobic surface is coated with amphiphilic molecules and/or polymers [12, 13]. Polymers can act as coordination sites for cadmium ion aggregation and protect QDs. Soluble polymers added during the synthesis have been used as a capping agent in the synthesis of CdS and CdSe nanoparticles, resulting in the well-controlled and uniform particle size of cadmium-rich nanoparticles [14]. Although the mixing of polymer and nanoparticles is not a novel scientific project [15], biocompatible polymer/QDs hybrid materials have shown great potential in the fields of biological and medical application [16, 17].

We recently synthesized cadmium sulfide semiconductor nanoparticles and coated them with sugar polymers [18, 19]. The experimental results revealed that polymer/QDs produced distinct dose-dependent effects. In this study, dextrin capped cadmium sulfide nanoparticles (CdS-Dx/QDs) were synthesized, characterized and subjected to a biocompatibility test. In order to determine material toxicity, three cell lines were incubated with CdS-Dx/QDs. The standard cell lines used in the viability assay were HepG2, HEK293 and HeLa. Cellular and subcellular uptake and bioimaging were also studied. We additionally reported in vivo fluorescence imaging, visualizing the fluorescence emission from CdS-Dx/QDs in tissues from rodents receiving single as well as multiple dosages.

## Results

### Synthesis and characterization of CdS-Dx nanoparticles

**Fig. 1:**
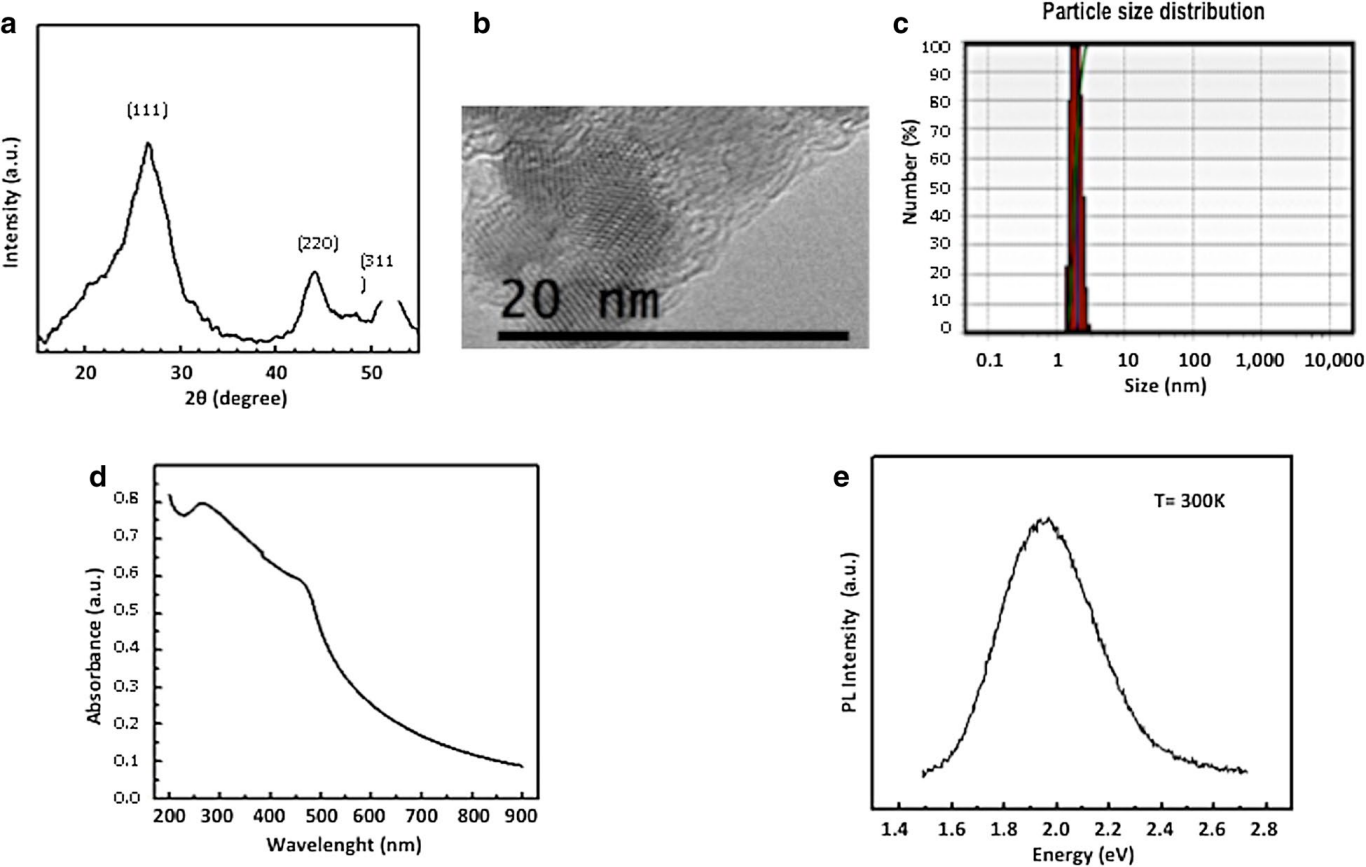
Quantum dot particles optical and structural characterization. **a** X-ray diffraction patterns of CdS-Dx/QDs. **b** HRTEM images of CdS-Dx/QDs. The nanoparticles appear agglomerated in the polymeric matrix of dextrin. A *close-up image* shows a *spherical* CdS nanoparticles with particle size in the range of 3–5 nm. **c** Size distribution of CdS-Dx/QDs. **d** UV–visible spectrum of size distribution of CdS-Dx/QDs. **e** Room temperature photoluminescence spectrum of CdS-Dx/QDs

X-Ray diffraction provides information about the crystalline structure, grain size and strain. Figure 1a shows the X-ray diffraction pattern of the CdS nanoparticles capped with dextrin. XRD peaks are found at *2* values of 26.5°, 43.96° and 52.13°, which refers to the diffraction from (111), (220) and (311) planes, respectively, of cubic zinc blend CdS rather than planes amorphous, which has only a single, very broad neighbor peak near the (111) line. The broadening of the diffraction peak provides information on crystallite size. As the width increases, particle size decreases and vice versa. Crystallite size was calculated using the Debye–Scherrer equation *d* = *0.8λ/βcosθ*, where *λ* is the wavelength of the X-ray radiation, *β* is the full width at half maximum (FWHM) of the (111) peak, and *θ* is the angle of diffraction. The average size of the CdS-Dx/QDs was determined to be of the order of 3 nm.

High-resolution transmission electron microscopy (HRTEM) was used to characterize the size and morphology of the CdS-Dx/QDs. Figure 1b shows the HRTEM micrograph, which displays random orientation and agglomeration of small CdS nanoparticles in the polymeric matrix of dextrin. The close-up TEM image shows uniform sphere-shaped nanoparticles with a particle size ranging between 3 and 5 nm. Figure 1c shows the size distribution of the CdS nanoparticles capped with dextrin, which were measured using Dynamic light scattering (DLS). As shown, the average size of CdS-Dx/QDs was about 3 nm, which is in good agreement with the crystallite grain size measurements from the XRD results and HRTEM.

We know UV–visible absorption spectroscopy is an efficient technique for monitoring the optical properties of quantum-sized particles. Figure 1d shows the absorption spectra of the synthesized CdS-Dx/QDs. Generally, the wavelength of the maximum peak absorption decreases as the particle size decreases because the quantum confinement of the photo-generated electron–hole pairs. The moderate changes in electronic absorption spectra of a CdS nanoparticle in aqueous solution, have been studied and a theoretical model has been proposed by Brus in order to determine particle size. Using Brus’s equation [20], we calculated a particle size of 3 nm, which is in good agreement with the crystallite grain sizes of the previously mentioned experiments. These optical characterization results show that, in general, the particles are isotropic in shape and size.

Photoluminescence (PL) refers to a material’s emission of light by any other process than blackbody radiation. Semiconductor nanoparticles have gained much attention because of their variety of narrow bandwidth emissions, which happen when nanoparticles are produced in sizes smaller than that of the typical Bohr radius. The PL spectra of CdS nanoparticles consist of green, yellow, and red band emission. Whereas green and yellow bands are related to band-edge emissions, the red band is related to defects on the nanoparticle surface. Figure 1e shows the room temperature PL spectrum of CdS-Dx/QDs. The PL spectrum of the CdS sample consists of a broad band emission centered in the 1.8–2.2 eV spectral region; the broadening of a luminescence band is associated to dispersion in the size of the nanoparticles.

### Effect of CdS-Dx/QDs on cell viability

**Fig. 2:**
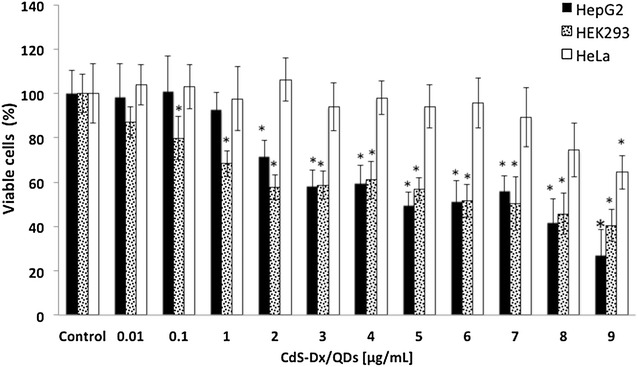
Effect of CdS-Dx/QDs on cell viability of human cell lines. **a** Effect of CdS-Dx/QDs on cell viability in HepG2, HEK293, and HeLa cells. Cells were exposed in cultured medium with different concentrations of nanoparticles for 24 h. Results are expressed as the percentage of cell viability as compared to control group. Data are presented as the mean ± SD of at least three independent experiments. *p < 0.05 as compared with control group

Figure 2 shows the effect of CdS-Dx/QDs on cell viability in human cell lines. As we can see, QDs decreased the number of hepatic cells (HepG2) by 25 % under concentrations of 2 μg/mL; 40–45 % under concentrations of 1–7 μg/mL; 63 % under 8 μg/mL, and 70 % under 9 μg/mL (p < 0.05). Kidney cells (HEK293), showed a significant reduction in the number of viable cells starting at concentrations of 0.1 μg/mL (20 %); above 1 μg/mL, this happened in a dose dependent manner (p < 0.05). Cervix cells (HeLa) did not show a significant decrease on cell viability; only a concentration of 9 μg/mL reduced cell viability by 32 % (p < 0.05).

### Characterization of cell death induced by CdS-Dx/QDs in cell lines

**Fig. 3:**
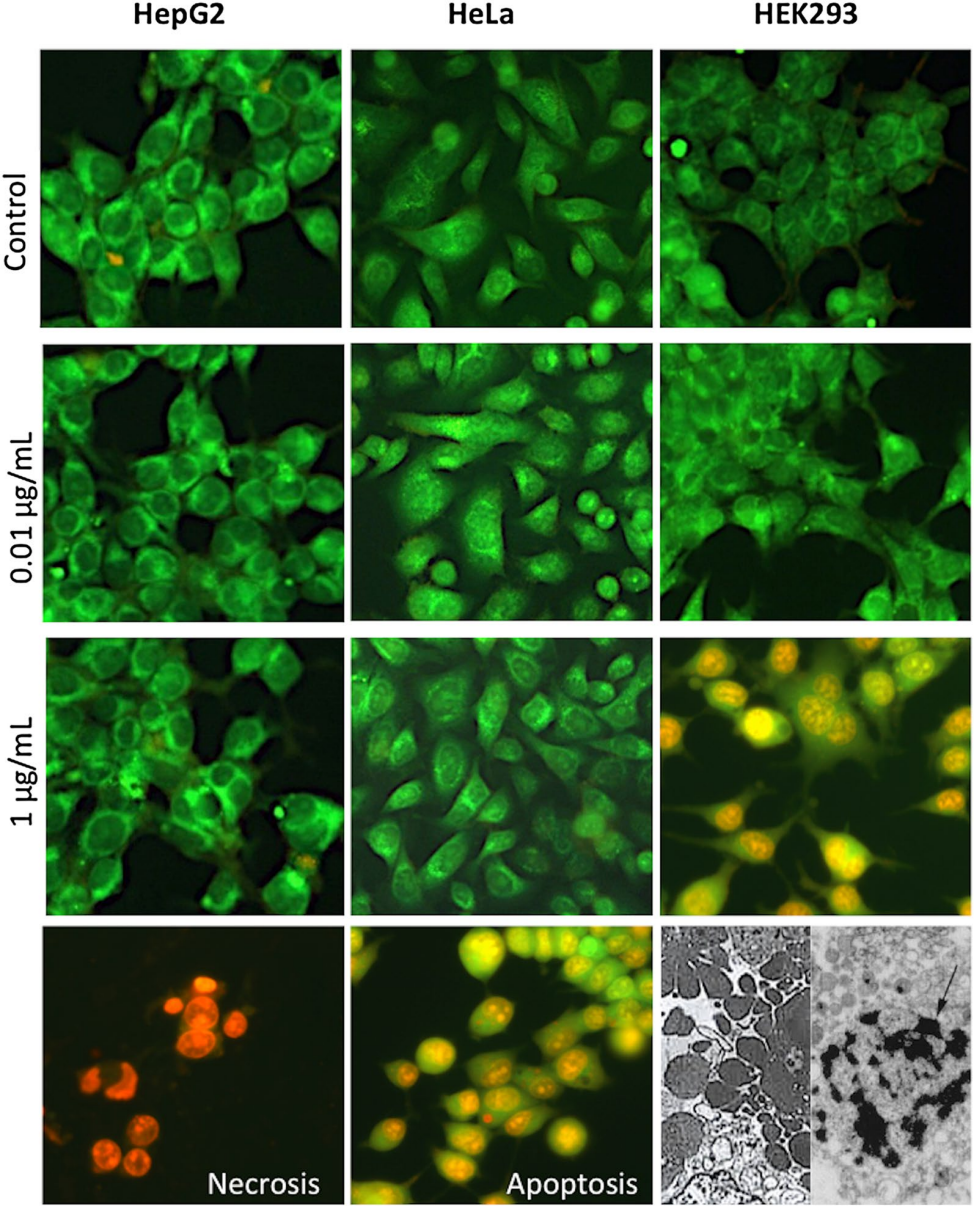
Cell death induced by CdS-Dx/QDs in human cell lines. Cells were treated with CdS-Dx/QDs (0.01 and 1 μg/mL) for 24 h and stained with AO/EtBr staining and analyzed using fluorescence microscopy (×100). Cells exposed to 1 µL/mL of 30 % H_2_O_2_ for 2 h were used as apoptosis; cells exposed to 100 °C for 5 min were used as necrosis; and non-treated cells were used as negative control. These are representative results of at least three independent experiments (n = 3)

Because HEK293 cells were the most sensitive to the cytotoxic effect of nanoparticles, we decided to carry out other assays in order to characterize the toxic effects of CdS-Dx nanoparticles in this cell line. Acridin orange/ethidium bromide (AO/EtBr) double staining was used to differentiate between apoptotic and necrotic cells. AO/EtBr staining revealed the absence of cell death in HepG2, HEK293, and Hela cells treated with 0.01 and 1 μg/mL CdS-Dx/QDs. Apoptotic cell death induction was observed in HEK293 cells when these were exposed to 1 μg/mL CdS-Dx/QDs for 24 h (Fig. 3). Ultra-structural analysis demonstrated that a significant portion of cells exposed to CdS-Dx nanoparticles exhibit the morphological features of apoptosis (membrane blebbing, formation of apoptotic bodies and chromatin condensation). No necrotic cells were found at 1 μg/mL concentrations.

#### Fluorescent microscopic visualization and quantitative analysis of the fluorescence of CdS-Dx/QDs in human cell lines

**Fig. 4:**
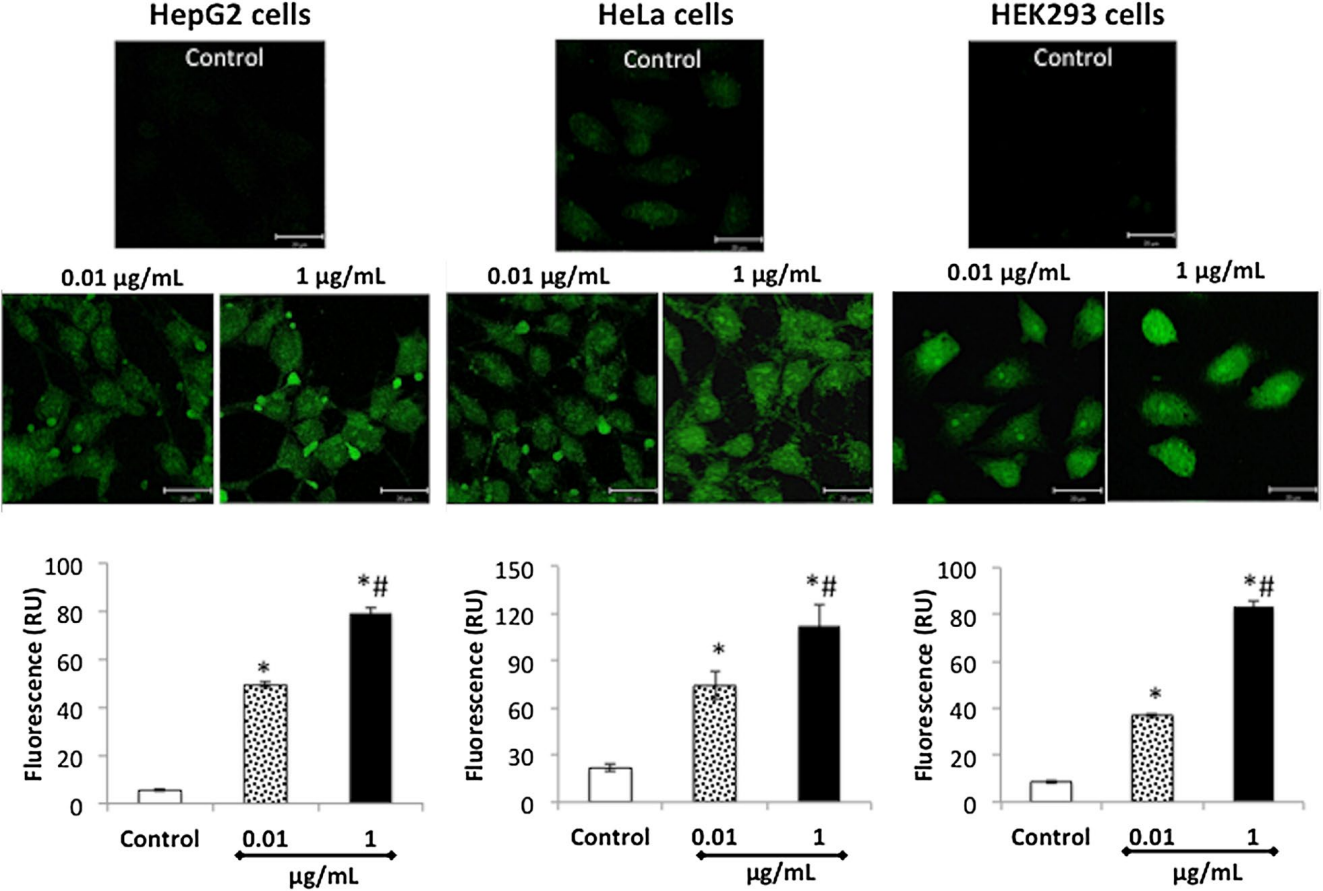
Fluorescent microscopic visualization and quantitation of CdS-Dx/QDs in human cell lines. Cells were incubated for 24 h with CdS-Dx/QDs (0.01 and 1 μg/mL), after which the free QDs were washed away, fixed in coverslips, and analyzed with a confocal microscope. Fluorescence images (*green*) showing cellular uptake of QDs in the cytoplasm of HepG2 and HeLa cells, and in the nucleus of HEK293 cells. *Scale bar* 20 μm. To measure the cellular uptake of CdS-Dx/QDs, cells were incubated with QDs (0.01 and 1 μg/mL) for 24 h. After this, the free QDs were washed away and the cells fixed. Cells were observed under fluorescence microscopy and analyzed using Image-Pro Insight 9 software. Data are presented as the mean ± SD of at least three independent experiments. *p< 0.05 as compared with control group. ^#^p < 0.05 as compared with 0.01 μg/mL

The fluorescent properties of CdS-Dx/QDs allow us to monitor their uptake and distribution directly. CdS-Dx/QDs uptake and bioimaging experiments were performed using a fluorescent microscope. For this, cells were incubated with 0.01 or 1 μg/mL CdS-Dx/QDs for 24 h and then washed to remove any unbound QDs. Interestingly, we were able to observe a uniform fluorescence pattern in most of the cells when examined under confocal fluorescence microscopy (Fig. 4). The quantitative analysis of this fluorescence showed a similar profile in all cell lines, and the increase in fluorescence happened in a dose-dependent manner (Fig. 4).

## *In vitro* cellular uptake localization of CdS-Dx/QDs in HEK293 and HeLa cells

**Fig. 5:**
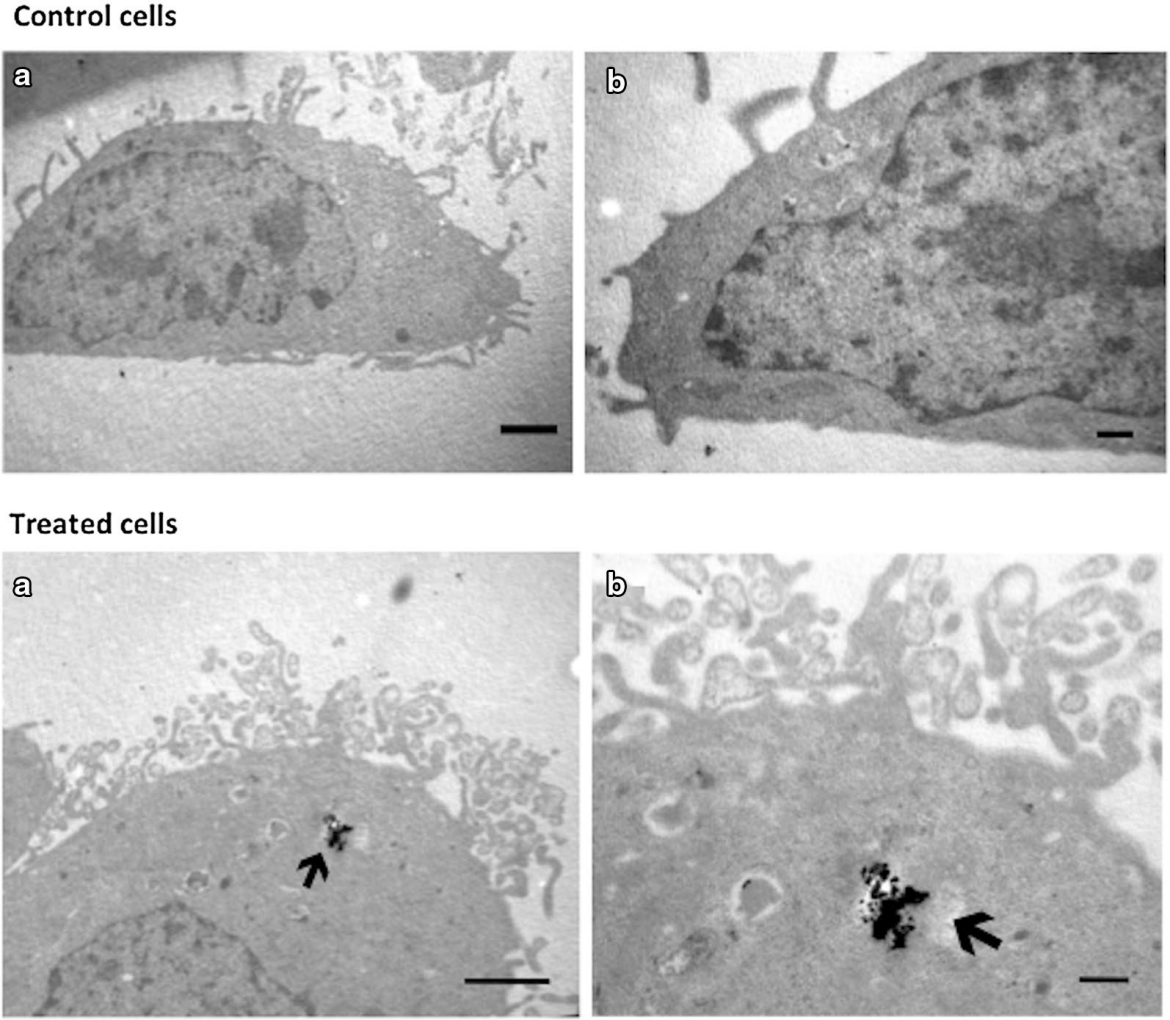
Intracellular location of NPs Cds particles in HeLa cells. Control cells. **a** TEM micrographs in low magnification of an untreated HeLa cell. **b** Correspond to high magnifications of cells. *Scales bars* for A = 1 μm; B = 200 nm. Treated cells **a** and **b** correspond to TEM micrographs of different magnifications of the same cell. Images correspond to the apical face of the epithelial cell. *Arrows* show vesicles with electrondense particles corresponding to the CdS-Dx/QDs. *Scale bars* for A = 1 μm and B = 200 nm

**Fig. 6:**
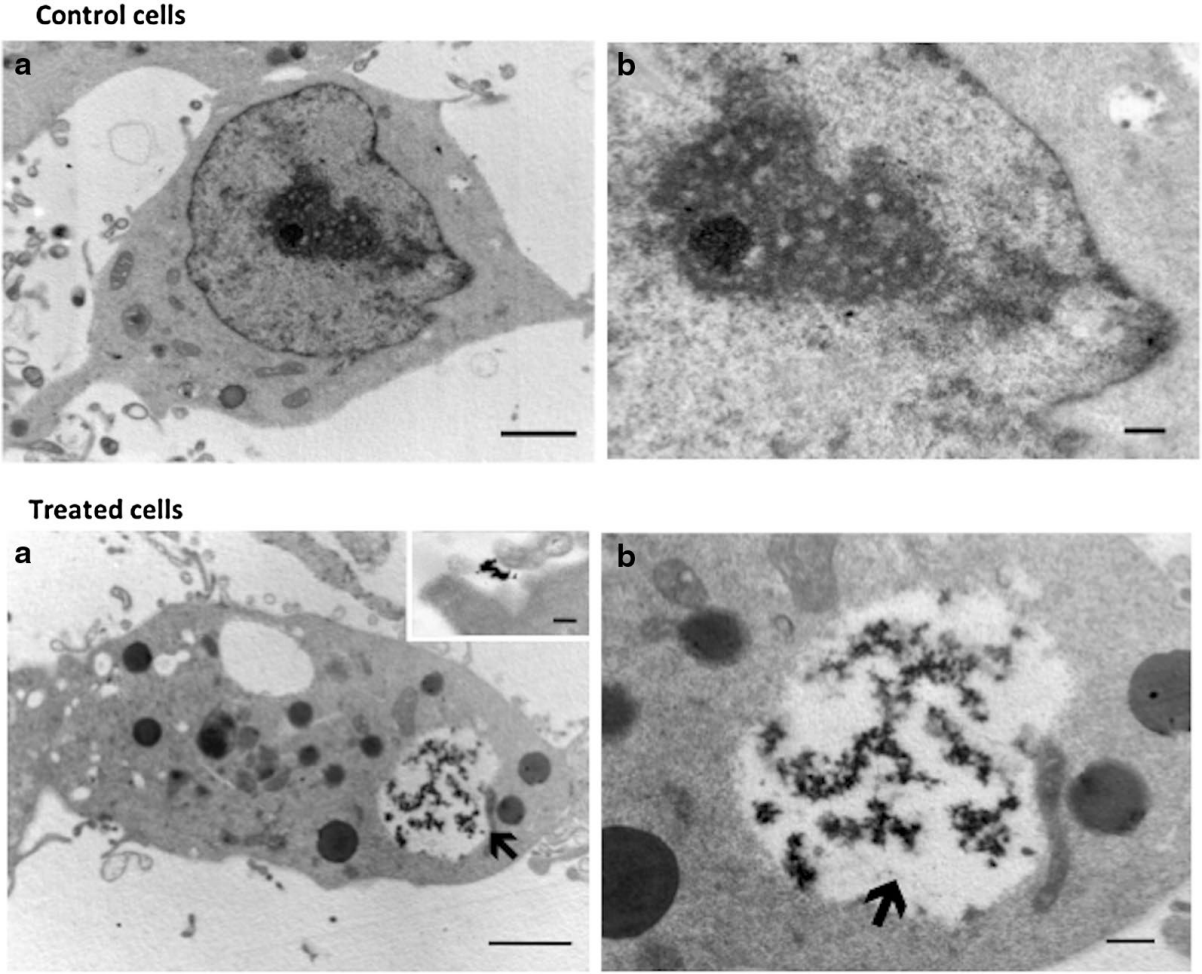
Intracellular location of the NPs Cds particles in HEK293 cells. Control cells. **a** Correspond a low magnification micrograph of a HEK293Q cells with its typical ameboid shape. **b** Correspond to high magnifications of cells. Scale bar in A = 1 μm; B = 200 nm. Treated cells. **a** Micrographs of HEK293 Q cells incubated with CdS-Dx/QDs. **b** Corresponds a magnifications of micrograph **a**. *Inset* in **a** correspond to the presence of the particles near to filopodial from a different cell. *Scale bar* in A = 1 μm; in B = 200 nm and in the *inset* in A = 100 nm

HEK293 and HeLa cells were incubated with 1 μg/mL CdS-Dx/QDs for 24 h and then washed to remove any unbound QDs. In untreated epithelial HeLa cells, the morphology was the usual for epithelial cells, including the presence of an apical face with abundant filopodia structures and a homogenous cytoplasm with some endocytic vesicles. The basal face of the cell was flat, with some membrane elongations probably related to the substrate by focal adhesions (Fig. 5). In contrast, HEK293 cells had ameboid shapes with vacuoles and showed cytoplasmic granules and abundant mitochondria (Fig. 6).

In presence of CdS-Dx/QDs, both HEK293 and HeLa cells showed the electrondense aggregates of the particles to be located within cytosolic vesicles (Figs. 5a, b, 6a, b). In HEK293 cells, the endocytic vesicles containing the particles were big and full with abundant aggregates of CdS-Dx/QDs. These cells presented numerous small vesicles, and it was occasionally possible to find particle aggregates attached to the extracellular face of the plasma membrane-mainly in zones with abundant filopodia (inset in Fig. 6a). In contrast, the endocytic vesicles of HeLa cells in which the CdS-Dx/QDs were found were usually small and apparently unique in the cytoplasm (Fig. 5a). The exposure of both cell types to the nanoparticles apparently did not modify their morphology.

### *In vivo* biodistribution and biocompatibility of CdS-Dx/QDs-fluorescence microscopy

**Fig. 7:**
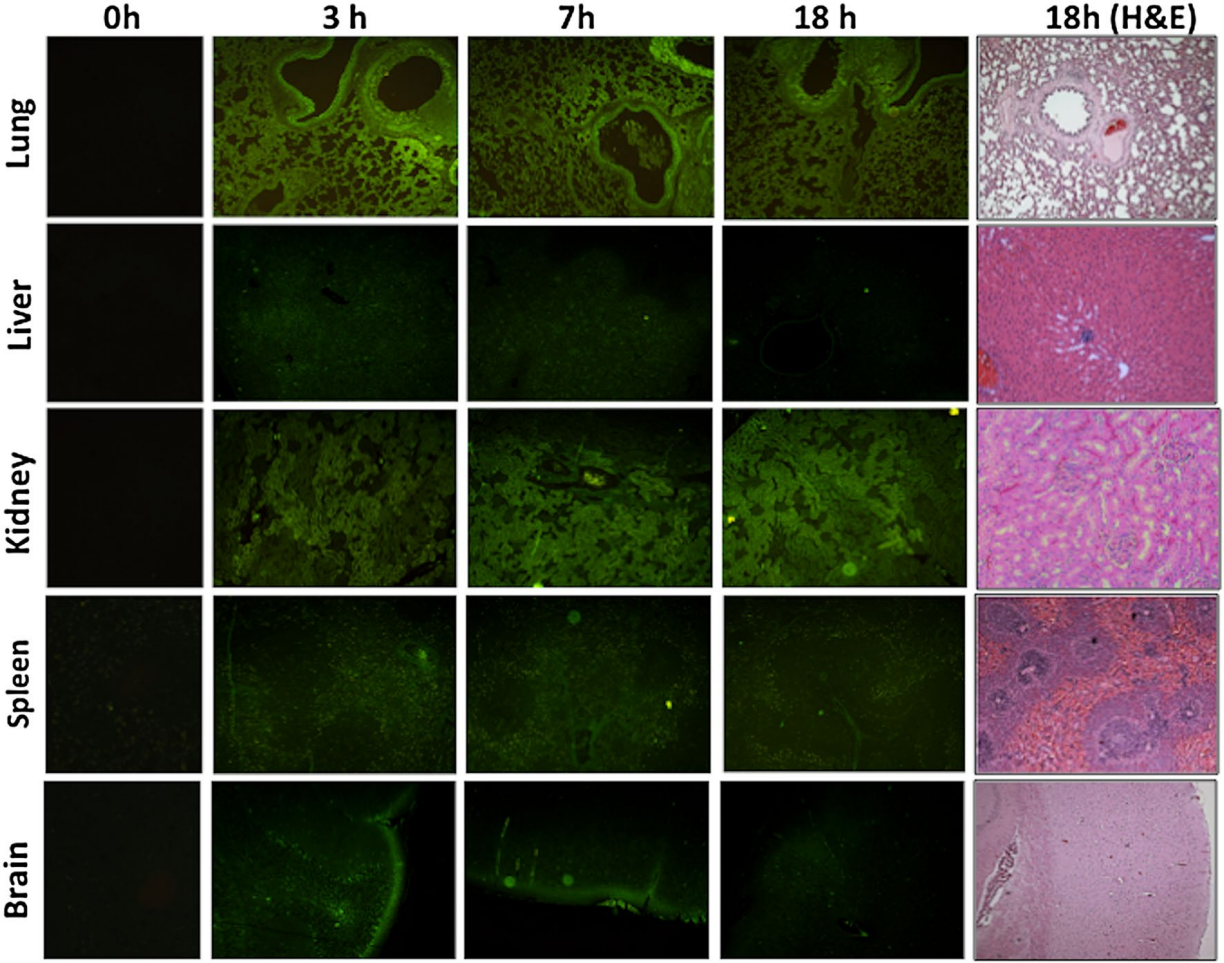
Fluorescence microscopic images showing the distribution and localization of CdS-Dx/QDs in tissue sections of rats after 3, 7 and 18 h i.p. administration. The images belong to lung, liver, kidney, spleen and brain and have a magnification of 10X. CdS-Dx/QDs fluorescence (*green*) in tissues showed their distribution and localization at different times. The *right column* shows tissues after 18 h of QDs administration; tissues were stained with Hematoxilin and Eosin (magnification ×10). The *left column* shows tissues from the control group observed under fluorescence microscopy

In order to obtain precise data regarding the tissue distribution of the QDs, we treated rats with CdS-Dx/QDs at 100 μg/kg. The unstained tissue samples were analyzed under a fluorescence microscope at 3, 7 and 18 h to see if the nanoparticles were homogenously distributed across all tissues or showed selectivity for a particular tissue. Figure 7 shows representative fluorescence images of the biodistribution of CdS-Dx/QDs in lung, liver, kidney, spleen and brain. Each tissue showed varying intensity of CdS-Dx/QDs fluorescence as time passed. In the lung, fluorescence was most intense at 3 h and decreased with time, while there was more distribution surrounding the blood vessels, bronchioles (smooth muscle, submucose and cartilage), and alveolar sacs (alveolar capillary barrier). The liver showed low fluorescence from 3 h through 18 h, indicating that the passage of nanoparticles through this organ was fast and they did not stay in it; however, nanoparticles surrounded the hepatic portal vein and branched into the hepatic artery, bile duct, and inside hepatocytes. In the kidney, fluorescence intensity increased with time, with the highest fluorescence found at 18 h. It mainly appeared in the proximal and distal convoluted tubule, being more reduced in glomeruli. In the spleen, fluorescence intensity decreased starting at 3 h and decreased more over time. The distribution was more evident in red pulp. It was clear that the CdS-Dx/QDs crossed the blood–brain barrier because we detected fluorescence in the brain. The presence of fluorescence was low in the cerebral cortex area and more intense in the plexiform layer. At 18 h, the degree of fluorescence was insignificant in this tissue. Intense fluorescence was observed in the cardiac and skeletal muscles, and very low fluorescence showed in the thymus and testis (data not shown). High intensity fluorescence in certain tissue sections indicated higher QDs internalization. The histopathological analysis did not reveal alterations in any of the studied tissues.

**Fig. 8:**
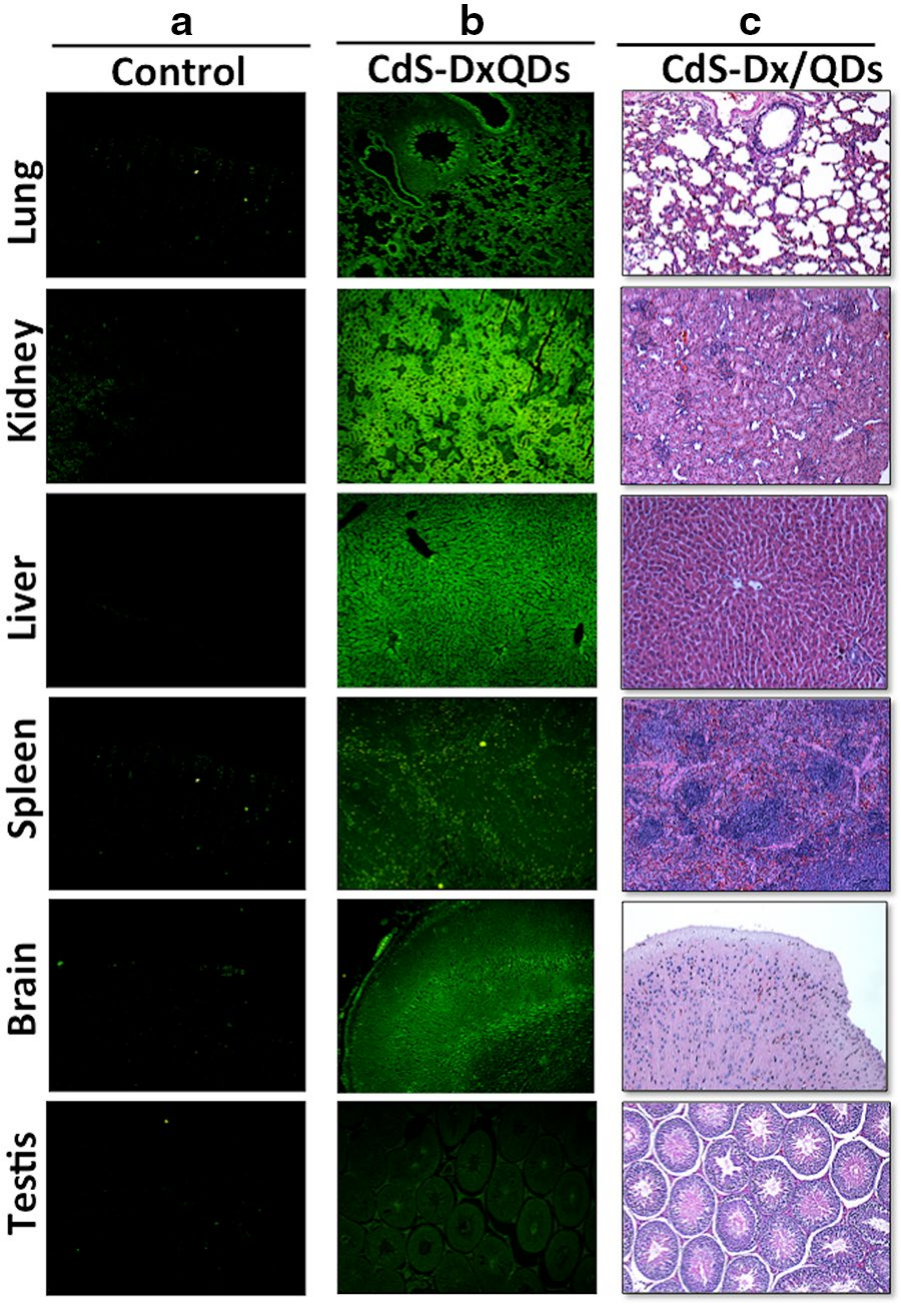
Fluorescence microscopic images showing the distribution and localization of CdS-Dx/QDs in tissue sections of rats after 7 days of treatment. The images belong to lung, kidney, liver, spleen, brain and testis (with a magnification of ×10). **a** Column shows tissues from the control group observed under fluorescence microscopy. **b** The distribution and localization of CdS-Dx/QDs (fluorescence *green*) in all tissues analyzed. **c** Histological sections from tissues after 7 days of QDs administration; tissues were stained with Hematoxilin and Eosin (magnification ×10)

In order to know the in vivo biocompatibility and toxicity of CdS-Dx/QDs, we treated rats with CdS-Dx/QDs at 100 μg/kg daily during 1 week. The unstained tissue samples were analyzed under a fluorescence microscope to see if the nanoparticles were homogenously distributed in all tissues and to determine their toxicity. Figure 8 shows representative fluorescence images of the biodistribution of CdS-Dx/QDs in lung, kidney, liver, spleen, brain and testis. The presence of differing fluorescence intensities due to the presence of CdS-Dx/QDs in each tissue was evident. In the lung, evident fluorescence surrounded the blood vessels, bronchioles (smooth muscle, submucose and cartilage) and alveolar sacs (luminal alveolar epithelium, smooth muscle and basement membrane). The kidney showed more intensity of fluorescence than any other organ. Fluorescence was detected mainly in the proximal and distal convoluted tubule and, to a lesser extent, in the glomeruli. The liver showed heightened fluorescence intensity, though this was mostly distributed around the hepatic portal venule, the branch of the hepatic artery, the bile duct and, to a lesser degree, in the hepatic parenchyma. In the spleen, fluorescence intensity was low when compared with other organs, and the distribution was more evident in red pulp. CdS-Dx/QDs clearly crossed the blood–brain barrier and blood-testis barrier because we detected fluorescence in both organs. Fluorescence was present in all layers of the cerebral cortex, but was heightened in the inner granular layer (IV) and the multiform cell layer (VI). Minor fluorescence was detected in the molecular layer (I). The testis showed intense fluorescence in Leydig cells and reduced intensity in the seminiferous tubule. A high uptake of CdS-Dx/QDs was observed in the cardiac muscle and skeletal muscle, and the lowest degree of fluorescence was detected in the thymus (data not shown). The histopathological analysis did not reveal alterations in any of the studied tissues after 1 week of exposure to CdS-Dx/QDs. Analyzed biochemistry parameters did not show any alterations as compared with control group (Table 1).

**Table 1:** Biochemistry parameters analyzed

Parameters	Control	CdS-Dx/QDs
Glucose (mg/dL)	97.96 ± 9.62	125.32 ± 21.56
Cholesterol (mg/dL)	44.31 ± 2.25	54.48 ± 12.83
Triglycerides (mg/dL)	71.69 ± 16.96	60.65 ± 8.78
Uric acid (mg/dL)	2.1 ± 0.14	1.8 ± 0.30
Creatinine (mg/dL)	0.46 ± 0.11	0.51 ± 0.05
Urea (mg/dL)	35.17 ± 2.2	35.52 ± 2.05
AST (U/L)	177.32 ± 18.36	198.12 ± 13.90
ALT (U/L)	55.91 ± 8.85	49.41 ± 13.45
ALP (U/L)	110.32 ± 9.43	127.38 ± 19.34

Data are mean ± SD

* p < 0.05 as compared with control group

## Discussion

This study successfully synthesized dextrin coated cadmium sulfide quantum dots. The results show that CdS-Dx/QDs can cause cytotoxicity in human cell lines; however, the toxicity differed significantly depending on the cell type and CdS-Dx/QDs concentration. We also showed evidence of the cellular uptake, intracellular localization, biodistribution and biocompatibility of CdS-Dx/QDs when used in vitro and in vivo. Due to their small size and physical resemblance to physiological molecules, QDs possess the capacity to revolutionize medical imaging, diagnostics, and therapeutics, as well as carry out functional biological processes [21, 22]. But they may also be toxic. Therefore, a detailed assessment of the biodistribution, biocompatibility and toxicity of QDs is crucial for sustainable biomedical applications and safe use.

Semiconductor nanocrystals are a new class of fluorescent biological labels. Originating in a confined quantum of the electron within the nanocrystal material, the fluorescence of QDs is unique as compared with traditional organic fluorophores. The incorporation of QDs into biological systems often requires strategies for the manipulation of the ligands as capping agent in order to improve the particle size and morphology of the resulting QDs. In particular, the dextrin polymer used as capping agent has polar groups of OH^−^ type. The hydroxyl groups spread over the surfaces and act as passivation centers for the stabilization of the CdS nanoparticles. These CdS-Dx/QDs obtained by aqueous synthesis are both disperse and stable and biocompatible—that is, compatible with living tissues or a living system by being neither toxic nor injurious or physiologically reactive.

The present study demonstrates that CdS-Dx/QDs produced different effects on human cell lines and caused cytotoxic effects depending on concentration. Indeed, some studies suggest that nanoparticles are not inherently benign and affect biological behavior at the cellular, subcellular, and protein levels [23–25]. QD toxicity, as with other nanoparticles, depends on multiple parameters as size, shape, concentration, charge, redox activity, surface coatings and mechanical stability [26]. The wide variation in these parameters in different experimental paradigms has posed a challenge to toxicological research in this area. To date, the literature on QD toxicity includes reports of numerous types of QDs with widely varying physicochemical parameters, making comparisons quite difficult [27, 28]. It is clear from this and other studies that surface coating is related to the toxicity experienced by cells, and this affects the level of toxic material released from the nanoparticles. The present study supports others that have indicated that different cell types have varying thresholds for QD-induced toxicity.

The development of better polymer/QDs materials that exhibit advantageous biocompatible and optical properties is an emerging research field. Here we synthesized CdS nanoparticles coated with a stable dextrin polymer and found a high cellular uptake in human cell lines and in vivo.

The internalization of CdS-Dx/QDs into human cells was confirmed by transmission electron microscopy, employed to characterize the uptake of CdS-Dx/QDs into cells. The images showed that CdS-Dx/QDs had different intracellular distributions, and the main reasons for this could be the different physiology of the cell lines and the cell related specificity of the internalization mechanism for QDs [29, 30]. Also, it should be noted that the uptake of QDs depends on their intrinsic properties, such as core material, shape, size, or charge [31]. The easy cellular uptake of CdS-Dx/QDs, as well as their even distribution and internalization into cells makes them suitable for drug delivery and use as intracellular fluorescent molecular tracers.

Although polymer/QDs are widely used in vitro, understanding how they move through the body will entail a breakthrough in this field, establishing the guidelines for in vivo bioapplication of polymer/QDs hybrid materials. This study assessed the biocompatibility and distribution of CdS-Dx/QDs in rodents. In order to be used in a biological environment, QDs need to be made hydrophilic. The incorporation of QDs into biological systems often requires strategies for the manipulation of the ligands bound to the QD surface in order to make them water-soluble and biocompatible—i.e., compatible with living tissue or a living system by being neither toxic nor injurious or physiologically reactive [32]. This study synthesized, characterized and analyzed the biocompatibility of QDs coated with dextrin. Dextrin is widely used in the industry due to its lack of toxicity. It is soluble in water and used as a binder in pharmaceutical products [33].

Several studies have looked at the in vivo biocompatibility and distribution of QDs. The nature of QDs in an in vivo system might be very different from that in an in vitro system because QDs can remain in the organism and directly interact with the living environment [34]. Currently, all of the literature has unanimously concluded that QDs show a preference for deposition in organs and tissues rather than remain circulating in the bloodstream [35]. The histological sections of tissue taken in this study showed a high degree of fluorescence under the fluorescence microscope and the distribution pattern of QDs at different times. The presence of high fluorescence in the tissue sections of most organs after 1 week of CdS-Dx/QDs exposure suggests that QDs are stable enough in vivo as well as biocompatible, given that they did not produce morphological or functional changes.

The journey of QDs to the desired site, however, is limited by a number of physiological barriers. These biological barriers are essential components of the body’s defense system and are designed to limit the penetration of foreign materials. The first barrier to intravenously administered QDs is the reticuloendothelial system, comprising the liver and spleen, which rapidly removes particles from the blood flow [36]. Our study evidenced the presence of CdS-Dx/QDs in the spleen in a single dose study, and in the liver after multiple doses. It is evident that QD distribution is determined by a number of interrelated physicochemical and biological factors, and an in-depth understanding of particle characteristics after injection into living subjects is needed to ensure the optimal performance of QDs as drug carriers.

It has been reported that QDs can enter the CNS through the BBB via systemic distribution [37]. Since the tight junctions in the BBB have 4–6 nm gaps, only QDs smaller than these sizes can pass through the inter-endothelial tight junctions, whereas most QDs pass through the endothelial cell plasma membranes by transcytosis [38]. The CdS-Dx/QDs synthesized in this study had a size of 3–5 nm and we found them in all layers of the cerebral cortex, which strongly suggests that CdS-Dx/QDs entered the brain via the BBB. CdS-Dx/QDs could be novel nanoparticles that enhance drug delivery efficacy across the BBB and facilitate the uptake of the QD-drugs in the brain. We also found CdS-Dx/QDs in testis, but no morphological changes. Further studies could discard toxic effects in these organs.

The high uptake of the CdS-Dx/QDs by the kidney and liver in the multiple dosage study suggests that the nanoparticles were retained, leading to efficient liver excretion and the kidney’s elimination of QDs from the body. The metabolism of QDs is yet another understudied aspect. The QDs cores do not appear to be subject to extensive enzymatic metabolism, but shells and coatings are. It has been suggested that the metabolic paths of QDs are closely correlated to their aggregation states and different metabolic paths have been associated after intravenous injection [39]. On the other hand, several studies suggest that the kidneys can remove QDs that are less than 5 nm. It has been observed that, after i.v. administration of CdSe/ZnS-QDs, only 10 and 40 % of the injected dose remained in the kidney and liver respectively, suggesting that only a fraction of the total QD dose passed through this route [40]. More comprehensive studies of potential excretion will therefore be critical to the development of QDs as nanopharmaceuticals.

Biological autofluorescence, arising from endogenous fluorophores, is an intrinsic property of cells and tissues. The autofluorescence properties of some tissues can serve as a useful diagnostic indicator in certain disease situations, where some proteins are accumulated (as liver fibrosis) [41]. In addition to intrinsic fluorescence of tissues, the autofluorescence may arise from the tissue-processing techniques, including fixation agents such as glutaraldehyde and embedding material such as paraffin. Although, autofluorescence could interfere with the emission of CdS-Dx QDs, nowadays there is various reagents used to eliminate AF, Sudan black B, NaBH_4_, CuSO_4_, Pontamine Sky Blue and mathematicals models, that have been proven effective in a range of tissues, including brain, liver, heart, and kidney, without adversely affecting the staining probabilities [42]. In present results we did not have problems with autofluorescence because we blocking the tissue samples fixed in formaldehyde with 1 mg/ml sodium borohydride. The absence of fluorescence in those tissue without CdS-Dx QDs was evident.

We believe that the information gathered in this study regarding the internalization, localization and distribution of CdS-Dx/QDs in cells is an important step forward in the use of QDs for biomedical research and theranostic applications.

## Conclusions

Our results indicate that CdS-Dx/QDs are cytotoxic at high concentrations. The cell uptake and intracellular distribution of CdS-Dx/QDs was different in the studied cell lines. Our in vivo results confirmed an effective cellular uptake and even distribution pattern of CdS-Dx/QDs in tissues. Fluorescence intensity was lower during short exposure periods and higher after 1 week. CdS-Dx/QDs were biocompatible and did not produce morphological changes in animal tissues. The CdS-Dx/QDs used in this study can be potentially used in bio-imaging applications.

## Methods

All chemicals were purchased from Sigma-Aldrich unless otherwise stated.

### Synthesis

Cadmium sulfide nanoparticles were prepared in aqueous solution. CdCl_2_ (5 mL, 0.02 M), KOH (10 mL, 0.5 M), NH_4_NO_3_ (5 mL, 0.5 M), and CS(NH_2_)_2_ (5 mL, 0.2 M) were added and the mixture was stirred and heated at 80 °C. Similar conditions were applied to maltodextrin with 3 % concentration. These solutions were slowly added into the flask and adjusted to pH 11 using a dilute solution of sodium hydroxide. The solution immediately turned a light yellow color, indicating the initial formation of a CdS nanoparticle. The temperature of the mixture was kept at 75 °C and maintained at this temperature for 60 min. The nanoparticles were separated from the chemical reaction by centrifugation at 6000 rpm during 60 min, and deposited in solid form from the solution; finally they were washed several times with deionized water and dried at 40 °C for 24 h. In the dextrin solution, the hydroxyl groups acted as stabilizer agents for the synthesized CdS nanoparticles. CdS nanoparticles have also been synthesized using starch and maltodextrin as a capping agent [17, 18].

### Nanoparticle characterization

The crystalline structure characterization of CdS-Dx/QDs was done using powder X-ray diffraction (XRD) spectrometer (D5000, Siemens, Germany). CdS-Dx/QDs were dispersed in ethanol and sonicated for 10 min, then placed on a cupper-net for evaluation using a Jeol2010 TEM (Jeol, USA). Transmission electron microscopy (TEM) imaging was used to determine the morphology and size of these nanoparticles. The particle size distribution was evaluated using dynamic light scattering on a Nanotrac Wave (Microtrac Inc, USA). The QDs dispersion was suitably diluted in deionized water at 25 °C. The UV–visible spectrum of CdS-Dx/QDs dispersed in deionized water was recorded using a Perkin-Elmer Lambda 25 spectrophotometer. The PL measurement was carried out by exciting the sample with the 325 nm line of a He-Cd laser at room temperature. The radiative emission from the sample was focalized toward the entrance slit of a HRD-100 Jovin-Ivon double monochromator with a resolution better than 0.05 nm, and detected with a Ag–Cs–O Hamamatsu photomultiplier with a spectral response in the 350–1000 nm range.

### Cell culture

HepG2 (hepatocellular carcinoma), HEK293 (Embrionic kidney), and HeLa (cervix adenocarcinoma) cell lines (ATCC, USA) were cultured in DMEM (GIBCO, USA), with 10 % FBS (GIBCO, USA) and 100 U/ml penicillin/100 µg/ml streptomycin (GIBCO, USA), in a humidified 5 % CO_2_ atmosphere at 37 °C.

### Cell viability assays

Cell viability and cell proliferation were determined using a MTT (methyl tetrazolium, Sigma Aldrich, USA) assay [43]. Regarding cell viability, HepG2, HEK293 and HeLa cells were seeded into a 96-well plate (10,000/well) and incubated for 24 h at 37 °C and 5 % CO_2_. The culture medium was replaced by a fresh one supplemented with different concentrations of CdS-Dx nanoparticles (0.01, 0.1, 1, 2, 3, 4, 5, 6, 7, 8 and 9 μg/mL) and incubated for 24 h. After treatment, the medium was gently removed and replaced with 20 μL MTT (5 mg/mL) and 150 μL of non-phenol-red medium, and incubated for 4 h. Medium from each well was discarded, followed by the addition of 200 μL DMSO and 25 μL Sorensen’s glycine buffer (glycine 0.1 M, NaCl 0.1 M, pH 10.5) to each well. When the formazan crystals were dissolved, the optical density was determined on a microplate reader (Bio-Rad) at a wavelength of 590 nm. Untreated cells served as a non-treated cell viability control. The results represented a percentage of the relative viability of cells vis-à-vis the untreated control. MTT results are presented as values relative to control values, expressed as percentages.

### Assessment of cell death by fluorescence microscopy

Assessment of cell death was carried out using the acridine orange and ethidium bromide staining assay as previously described [44]. Briefly, the HepG2, HEK293 and HeLa cells were seeded into 6-well plate (250,000/well) and incubated for 24 h at 5 % CO_2_ and 37 °C. Culture medium was replaced with fresh media containing CdS-Dx/QDs at 0.01 and 1 μg/mL and the cells were then incubated for another 24 h. After thorough washing with DPBS, 250 µL of a mixture of 100 µg/mL acridine orange/100 µg/mL ethidium bromide (Sigma Aldrich, USA) was added to the each well. The cells were then incubated at room temperature for 10 s and observed under a fluorescence microscope. Images of fluorescently stained cells were photographed with an Olympus digital camera. The data represents the average number of live, apoptotic or necrotic cells over at least 15 images for each treatment. Cells incubated in culture medium were used as a non-treated control. 1 µL/mL of 30 % H_2_O_2_ served as apoptosis control and smashed cells were used as necrosis control. Cells were categorized as healthy (green fluorescent cells without any nuclear staining), apoptotic (condensed or fragmented orange red nucleus) or necrotic (orange red, “apparently normal” or patchy nucleus).

#### Fluorescent microscopic visualization and quantitative analysis of fluorescence of CdS-Dx/QDs in human cell lines

HepG2, HEK293 and HeLa were used to verify the selective uptake of CdS-Dx/QDs. The cells (1 × 10^5^) were seeded onto 12 mm sterile coverslips in a 6-well plate. The cells were cultured for 24 h, washed thrice with PBS, and then incubated with CdS-Dx/QDs (0.01 and 1 μg/mL) for 24 h. After washing thrice with cold PBS, the cells were fixed for 20 min in 200 µL of 4 % paraformaldehyde. After a time, the cells were washed again with PBS buffer. The coverslip with fixed cells was topped with a glass slide with a drop of 10 µL of 50 % glycerol/PBS (v/v) and placed above the objective on a confocal microscope (Nikon Al, Nikon, Japan). CdS-Dx/QDs were excited with a 488 nm laser, and their signals were collected from 515 nm. In order to measure the cellular uptake of CdS-Dx/QDs, the coverslip with fixed cells was observed under fluorescence microscopy and analyzed using the Image-Pro Insight 9 software (Media Cybernetics Inc.).

#### Procedure for cells exposed to CdS-Dx/QDs for TEM

HeLa and HEK293 cells grown in Tc-treated culture dishes (60 mm in diameter) were incubated with fresh 1 μg/ml CdS-Dx/QDs for 24h. Extracellular CdS-Dx/QDs were discarded after thorough washing with PBS. Processing for TEM was done according to Muñiz et al. [45]. Briefly, cells were fixed with 2.5 % glutaraldehyde for 2 h at RT, followed by thorough washings with PBS. Cells were scrapped off and then centrifuged at 2000 rpm to form a cell pellet. Cells were postfixed for 1 h in 1 % OsO4 at 4 °C. Samples were rinsed with PBS and then gradually dehydrated in increasing concentrations of ethanol and finally embedded in Spurr’s resin and polymerized at 60 °C for 48 h (Electron Microscopy Sciences, Washington, DC). Thin sections mounted on grids were obtained using a Reichert Jung ultramicrotome (Reichert Jung, Austria). They were afterwards stained with uranyl acetate and lead citrate. Grids were viewed in the TEM.

### Analysis of in vivo biodistribution and biocompatibility of CdS-Dx/QDs

The Wistar rat was selected as the model for the biocompatibility study of CdS-Dx/QDs. All animals were kept in an animal house for 12 h day/night cycle for 2 months. All animals were kept in stress-free, hygienic, and animal-friendly conditions, housed in a temperature and humidity controlled environment, and allowed food (Standard Purina Chow Diet, Mexico) and water ad libitum. The experiments were conducted in accordance with the Guide for the Care and Use for Laboratory Animals [46].

Healthy Wistar rats (8–10 weeks old) were selected randomly and divided into two groups: one group received a single dose of CdS-Dx/QDs and the second group received CdS-Dx/QDs in multiple doses; each group had its respective control group. For the single dose assay, 15 animals were treated with a single dose of CdS-Dx/QDs at 100 μg/kg body weight i.p., in 300 μL PBS. Five animals were sacrificed at 3, 7 and 18 h. Animals were observed for signs of toxicity. Signs recorded during acute toxicity included: motor activity, anaesthesia, tremor, arching, and rolling, clonic convulsions, ptosis, tonic extension, lacrimation, exophthalmos, pilo-erection, salivation, depression, ataxia, sedation, hypnosis, cyanosis and analgesia. Behavior parameters, death, weight, and the amount of water and feed were analyzed.

For a multiple dose assay, animals were treated with CdS-Dx/QDs at 100 μg/kg i.p. daily for 1 week, in 300 μL PBS. Control groups were injected with 300 μL PBS. Toxicity indices consisted of daily clinical observations, body weight, food consumption, clinical pathology, organ weights, and histopathology.

After treatment, animals were fasted overnight and blood samples were obtained from the heart following anesthesia with ether. Tissues collected at necropsy were preserved in 10 % neutral-buffered formalin fixative and were processed for routine histologic examination. For fluorescence analysis, tissue samples were not stained with H&E stain. For reduce the autofluorescence in tissue rinse many times the tissue with 1 mg/ml solution of sodium borohydride, as blocking agent. Serum was separated by centrifugation at 3000 rpm for 15 min. Biochemical parameters of serum enzyme activities of alanine aminotransferase (ALT), aspartate aminotransferase (AST), alkaline phosphatase (ALP), glucose, cholesterol, triglyceride (TG), urea, creatinine and uric acid from animals that received multiple doses of CdS-Dx nanoparticles were measured using a commercial reagent kit (ELITech, Mexico).

### Statistical analysis

The data were represented as the mean ± SD of 3 independent experiments conducted in octuplicate. The data was statistically analyzed using the SPSS 10.0 software (SPSS Inc., Chicago, IL, USA), the *ttest*, and ANOVA. Differences were considered significant if the *P* value was less than 0.05.

## Abbreviations

QD: quantum dot
Cd++: cadmium
CdS: cadmiun sulfide
CdS-Dx: cadmium sulfide-dextrin
XRD: X-ray diffractometer
TEM: transmission electron microscopy
AO/EtBr: acridine orange/ethidium bromide
DMEM: dulbecco’s modified eagle medium
MTT: tetrazolium salt
BBB: blood brain barrier
CNS: central nervous system
